# London's Record Epidemic

**Published:** 1921-03-12

**Authors:** 


### London's Record Epidemic.

### ACHIEVEMENT OF THE METROPOLITAN ASYLUMS BOARD.

The infectious disease outbreak of last year was, from the point of view of numbers concerned, the biggest epidemic which the Metropolitan Asylums Board has ever been called upon to deal with. In November there were 8,669 patients in hospital, 1,511 more than the highest number the Board had previously ever had to deal with. The cases under treatment included scarlet fever, 5,664; diphtheria, 2,594; enteric fever, 15; tuberculosis, 334; and other diseases, 62. From July cases were admitted at the rate of 100 to 120 a day. The multitude of diphtheria cases added to the difficulties of the situation, as it is only possible to transfer to the convalescent hospitals a comparatively small percentage of such patients, with the result that the great majority have to be retained in town hospitals until they are ready to be discharged. Up to the end of the year the daily number of admissions to the acute hospitals exceeded 150 on no less than sixty-four occasions, and on sixteen out of this number they exceeded 200, the highest number of any one day being 244 on November 5. In view of the great difficulty experienced last autumn and winter in securing the nursing staff needed for the full working of the various hospitals, the matrons were asked in the early part of July to take all practicable steps to obtain staff to meet requirements during the rise that was then beginning. As, however, it appeared that nurses were not forthcoming in anything like the numbers required in response to the usual efforts to obtain them, steps were taken to advertise vacancies systematically and extensively in the usual nursing newspapers, and also in newspapers circulating in all parts of the country. As a result, over five thousand letters of inquiry were received and answered, and some 800 nurses were engaged by the matrons of the several hospitals. The approximate cost of the advertising campaign was £900, and considering the success attained the Board thinks the money well spent. Nevertheless, the number of nurses engaged by no means satisfied the needs, and it was found absolutely necessary to supplement them by employing nurses from nursing institutes to a maximum of 197. Approval was also given to the working of overtime by the staff wherever, by this, it was found that a larger number of patients could be accommodated in the various hospitals.

With a view to utilising the large resources of Joyce Green Hospital, Dartford, in the early stages of the rise, arrangements were made by which on several days a week a number of acute cases of scarlet fever were removed from their homes to South Wharf, Rotherhithe, and conveyed either later in the same day, or the following day, to Long Reach (for transfer by ambulance tram to the adjacent Joyce Green Hospital) by one of the Board's ambulance steamers. This steamer, on return, brought up recovered patients. This arrangement was only rendered possible by the fortunate absence of any small-pox cases. The report refers to many such difficulties which had to be overcome by reason of three hospitals having been in military possession. In view of the urgent demand for accommodation, and also of the mildness of the prevailing types of disease, the Board was able to put in extra emergency beds in the wards during the period of extreme urgency, and so largely add to the resources. The furniture and equipment was obtained through the Board's Contract Committee, large purchases being made from the military authorities.

When, owing to extreme pressure, it was doubtful if beds could be provided for all cases immediately on application, special efforts were made to admit every diphtheria patient at once on account of the graver nature of the disease, and these efforts were entirely successful except on three occasions, when ten cases were held over from one evening till the following morning. This policy entailed in many instances the conversion of scarlet fever wards to diphtheria purposes. The vast majority of the scarlet fever cases throughout the epidemic were likewise removed at once, but at the busiest times delay took place in removing some of the cases. However, no case was refused admission.

